# *Pimenta dioica* (L.) Merr. Bioactive Constituents Exert Anti-SARS-CoV-2 and Anti-Inflammatory Activities: Molecular Docking and Dynamics, In Vitro, and In Vivo Studies

**DOI:** 10.3390/molecules26195844

**Published:** 2021-09-27

**Authors:** Heba A. El Gizawy, Sylvia A. Boshra, Ahmed Mostafa, Sara H. Mahmoud, Muhammad I. Ismail, Aisha A. Alsfouk, Azza T. Taher, Ahmed A. Al-Karmalawy

**Affiliations:** 1 Department of Pharmacognosy, Faculty of Pharmacy, October 6 University (O6U), October 6 City, Giza 12585, Egypt; hebaelgizawy@o6u.edu.eg; 2 Department of Biochemistry, Faculty of Pharmacy, October 6 University (O6U), October 6 City, Giza 12585, Egypt; Sylviaazmy@o6u.edu.eg; 3 Center of Scientific Excellence for Influenza Viruses, National Research Centre (NRC), Dokki, Giza 12622, Egypt; ahmed_elsayed@daad-alumni.de (A.M.); sarahussein9@yahoo.com (S.H.M.); 4 Department of Pharmaceutical Chemistry, Faculty of Pharmacy, The British University in Egypt, Al-Sherouk City, Cairo-Suez Desert Road, Cairo 11837, Egypt; m.ismail.800@gmail.com; 5 Department of Pharmaceutical Sciences, College of Pharmacy, Princess Nourah bint Abdulrahman University, Riyadh 84428, Saudi Arabia; aaalsfouk@pnu.edu.sa; 6 Department of Pharmaceutical Organic Chemistry, Faculty of Pharmacy, Cairo University, Cairo 11562, Egypt; 7 Department of Pharmaceutical Organic Chemistry, Faculty of Pharmacy, October 6 University (O6U), October 6 City, Giza 12585, Egypt; 8 Department of Pharmaceutical Medicinal Chemistry, Faculty of Pharmacy, Horus University-Egypt, New Damietta 34518, Egypt

**Keywords:** *Pimenta*, rutin, anti-SARS-CoV-2, docking and dynamics simulations, in vitro and in vivo studies

## Abstract

In response to the urgent need to control Coronavirus disease 19 (COVID-19), this study aims to explore potential anti-SARS-CoV-2 agents from natural sources. Moreover, cytokine immunological responses to the viral infection could lead to acute respiratory distress which is considered a critical and life-threatening complication associated with the infection. Therefore, the anti-viral and anti-inflammatory agents can be key to the management of patients with COVID-19. Four bioactive compounds, namely ferulic acid **1**, rutin **2**, gallic acid **3**, and chlorogenic acid **4** were isolated from the leaves of *Pimenta dioica* (L.) Merr (ethyl acetate extract) and identified using spectroscopic evidence. Furthermore, molecular docking and dynamics simulations were performed for the isolated and identified compounds (**1**–**4**) against SARS-CoV-2 main protease (Mpro) as a proposed mechanism of action. Furthermore, all compounds were tested for their half-maximal cytotoxicity (CC_50_) and SARS-CoV-2 inhibitory concentrations (IC_50_). Additionally, lung toxicity was induced in rats by mercuric chloride and the effects of treatment with *P. dioca* aqueous extract, ferulic acid **1**, rutin **2**, gallic acid **3**, and chlorogenic acid **4** were recorded through measuring TNF-α, IL-1β, IL-2, IL-10, G-CSF, and genetic expression of miRNA 21-3P and miRNA-155 levels to assess their anti-inflammatory effects essential for COVID-19 patients. Interestingly, rutin **2**, gallic acid **3**, and chlorogenic acid **4** showed remarkable anti-SARS-CoV-2 activities with IC_50_ values of 31 µg/mL, 108 μg/mL, and 360 µg/mL, respectively. Moreover, the anti-inflammatory effects were found to be better in ferulic acid **1** and rutin **2** treatments. Our results could be promising for more advanced preclinical and clinical studies especially on rutin **2** either alone or in combination with other isolates for COVID-19 management.

## 1. Introduction

COVID-19 is a pandemic disease introduced by a novel coronavirus strain. Previously, this disease was known as ‘2019-nCoV’ which stands for ‘2019 novel coronavirus’. The causative virus in COVID-19 infection belongs to the Coronaviridae family which is a group of positive-sense, single-strand RNA viruses. Coronaviruses are considered a big family that is usually non-pathological to humans or may cause very mild symptoms such as the common cold. Some strains may infect humans while others may infect animals. Cross-over of the virus from an animal carrier to a human are seldom, but, in very rare cases, the coronavirus that causes infection to an animal is mutated and can transfer to humans. This was reported in Middle East Respiratory Syndrome (MERS) and Severe Acute Respiratory Syndrome (SARS) [1,2]. By 1 August 2021, the virus had infected over 198.9 million people worldwide, and the number of deaths had totaled more than 4.2 million [3].

Researchers are searching to find a potential cure for the Severe Acute Respiratory Syndrome Coronavirus-2 (SARS-CoV-2) disease, some of these trials include herbal medicine. The immune status of COVID-19 patients has a great influence on the disease severity and complexity. Therefore, herbal products with an immunomodulatory effect could have a positive impact on COVID-19 patients as preventive and therapeutic agents [4,5]. Although COVID-19 vaccination has a great influence on controlling the disease, scientists are once again warning that vaccines alone will not end the pandemic. People need to improve their immune system the main line of defense against the virus, adhere to basic preventative measures to keep physical distancing, wearing masks, and hand washing [6].

Moreover, patients infected with SARS-CoV may suffer from acute respiratory distress syndrome (ARDS). It is common in COVID-19 severe cases. The viral immunological response produces cytokine storm in severe infections which are characterized by an apparent increase in cytokines (IL-2, 7, and 10), IFN-γ-induced protein-10 (IP10), granulocyte colony-stimulating factor (GSCF), monocyte chemoattractant protein-1(MCP1), and tumor necrosis factor-α (TNF-α). The previously mentioned factors may have seriously damaging outcomes which require the administration of strong anti-inflammatory drugs to save lives and decrease the rate of mortality [7].

*Pimenta dioica* (L.) Merr is an aromatic spice plant that belongs to the family Myrtaceae; which includes around 150 genera and 3300 species that spread commonly in the tropics. They have leathery evergreen leaves with oil glands. Some members are of economic importance such as eucalyptus, guava, and clove. *P. dioica* is known as allspice or Jamaica Pepper due to its unique aroma blend which is used commonly in traditional medicine due to its volatile oil composition. *P. dioica* is grown in Jamaica, Central America, West India, and Mexico. Leaves of *P. dioica* are widely used as food ingredients in many countries. Furthermore, it has therapeutic effects to treat some diseases as a herbal medicine [8]. Many phenolic compounds such as ericifolin, eugenol, gallic acid, and quercetin [9] were isolated from *P. dioica* berries [10,11]. Extracts obtained from *Pimenta* stems have been confirmed to have good antimicrobial activity in addition to analgesic and anti-inflammatory properties. The good anti-inflammatory effects are not only attributed to the inhibition of the mediators that promote inflammation but also due to reduction of the inflammatory edema [12].

Ferulic acid is a ubiquitous phenolic compound of plant tissues constituting a bioactive ingredient of many foods. It displayed antioxidant and cytoprotective effects and could have a potential therapeutic effect in many diseases including cardiovascular diseases, diabetes mellitus, cancer, Alzheimer’s disease, and skin diseases [13].

Rutin, a flavonoid glycoside, has different protective effects against liver injuries in rats and hemodynamic alteration through antioxidant activities associated with ischemia and reperfusion. It has an inhibitory effect on the peroxidation of membrane lipids and oxidative stress diseases [14,15].

Chlorogenic acid has many physiological functions, such as neuroprotection, neuro-nutrition, antioxidant and anti-inflammatory. It has antibacterial and antifungal properties [16].

Furthermore, gallic acid has demonstrated a variety of biological activities including ameliorative, anti-inflammatory, and antitumor activities [17].

The main protease (Mpro) enzyme of SARS-CoV-2 is the one that helps in the conversion of its polypeptides into functional proteins and is responsible for its transcription and replication as well [18]. Accordingly, targeting the Mpro of SARS-CoV-2 appears to be very promising for the management of the COVID-19 pandemic [19].

Computational drug design methods have become very reliable and widely used in drug discovery processes nowadays [20,21,22]. Molecular docking and molecular dynamics simulations help scientists greatly in the fast-track discovery of new drug candidates [23,24,25].

**Figure 1:**
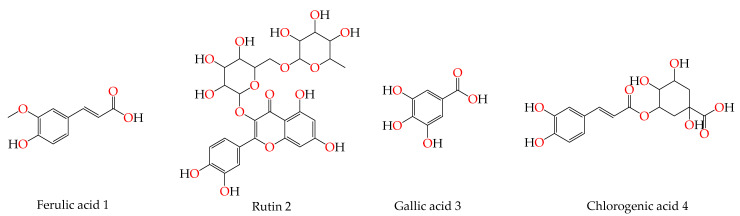
Chemical structures of the isolated bioactive phenolic compounds (ferulic acid **1**, rutin **2**, gallic acid **3**, and chlorogenic acid **4**) from the leaves of *Pimenta dioica* (L.) Merr.

Based on the aforementioned information concerning the essential role of Mpro in SARS-CoV-2 replication, besides the reported antiviral activity of *P. dioica* (L.) Merr [10], herein, and as an extension to our previous promising research targeting SARS-CoV-2 [26,27,28,29,30,31,32,33,34,35], we decided to examine the Mpro inhibitory activities of the four isolated compounds (ferulic acid **1**, rutin **2**, gallic acid **3**, and chlorogenic acid **4**) -depicted in Figure 1—using molecular docking and dynamics simulations. Moreover, to confirm our findings, we evaluated the anti-SARS-CoV-2 activities of the isolates (**1**–**4**) using MTT cytotoxic and inhibitory concentration 50 (IC_50_) determination assays and examined their anti-inflammatory effects on different cytokines and genetic markers as well.

## 2. Results and Discussion

### 2.1. Chemistry

The full chemistry data of the isolated compounds were represented in the Supplementary Data (SI1).

### 2.2. RP-HPLC Analysis

RP-HPLC analysis of the aqueous leaves extract of *P. dioica* revealed the presence of 13 components. Major phenolics were gallic acid (13,496.53 µg/g) and chlorogenic acid (3897.96 µg/g), meanwhile, the major flavonoid detected was naringenin (7514.91 µg/g) (Table 1). Chromatograms revealed the absence of vanillin, kaempferol, and cinnamic acid.

### 2.3. Docking Studies

The N3 co-crystallized inhibitor of SARS-CoV-2 Mpro became stabilized inside its binding site in an asymmetric form. Molecular docking of the isolated four compounds (ferulic acid **1**, rutin **2**, gallic acid **3**, and chlorogenic acid **4**) of *P. dioica* (L.) Merr extract together with the N3 inhibitor (docked, 5) into the main protease binding site was performed. Their binding scores were promising with the following descending order: N3 inhibitor (docked, 5) > rutin (**2**) > chlorogenic acid (**4**) > ferulic acid (**1**) > gallic acid (**3**). Moreover, their interactions with the receptor amino acids were compared to that of the docked N3 inhibitor (**5**) as well (Table 2).

Analyzing the docking results indicated that both rutin (**2**) and chlorogenic acid (**4**) showed the best scores and interactions inside the binding site of SARS-CoV-2 Mpro. Rutin (**2**) achieved a binding score of −9.19 kcal/mol, which seems to be nearly the same as the docked N3 inhibitor (**5**) which achieved −9.22 kcal/mol. It formed five hydrogen bonds with His163, Glu166, Phe140, and Cys145 amino acids of the pocket. Furthermore, it bound His41 through a hydrogen-pi bond. On the other hand, chlorogenic acid (**4**) achieved a promising binding score of −7.18 kcal/mol, and bound Asn142, His164, Arg188, and Met165 amino acids of the pocket through the formation of four hydrogen bonds as well (Table 2 and Table 3).

Based on the above, it can be concluded that the superior binding abilities of rutin (**2**) and chlorogenic acid (**4**) are based on two important principles (Figure 1 and Table 3):

(a) Their larger size compared to both ferulic acid (**1**) and gallic acid (**3**) of smaller sizes enabled them to occupy the large pocket of SARS-CoV-2 Mpro (similar to its co-crystallized polypeptide inhibitor, N3).
(b) The presence of polyphenolic moieties and polyhydroxy groups in the chemical structures of the aforementioned tested compounds (rutin **2** and chlorogenic acid **4**), besides the carboxylic acid group in chlorogenic acid **4** with nearly similar distances compared to the main functional groups of the N3 inhibitor. The previously mentioned functional groups with their described distances enabled them to bind the crucial amino acids inside the branched large pocket of SARS-CoV-2 Mpro.

The discussed docking findings of the isolated four compounds (ferulic acid **1**, rutin **2**, gallic acid **3**, and chlorogenic acid **4**) of *P. dioica* (L.) Merr extract compared to the N3 inhibitor (docked, 5) into the main protease binding site, built a promising good idea concerning the binding affinities and the expected intrinsic activities concerning the isolated tested compounds towards the SARS-CoV-2 main protease.

Moreover, this study proposed the aforementioned tested compounds (**1**–**4**), especially rutin (**2**) and chlorogenic acid (**4**) as potential SARS-CoV-2 Mpro inhibitors which are recommended to be further examined by more in vitro and also in vivo studies to gain an effective therapy targeting SARS-CoV-2 pandemic as soon as possible.

### 2.4. Molecular Dynamics Simulations

Molecular dynamics (MD) simulations were carried out to compare the binding stability of the four compounds (ferulic acid **1**, rutin **2**, gallic acid **3**, and chlorogenic acid **4**) of *P. dioica* (L.) Merr extract inside the Mpro binding site. MD run with the co-crystallized N3 ligand 5 was carried out to act as a control simulation. Another MD run with the docked N3 ligand 6 was conducted in order to compare its dynamics to the reference co-crystallized ligand and account for the reliability of the docking results and act as a docking validation in addition to RMSD measurement. MD was performed for 100 ns at NPT.

**Figure 2:**
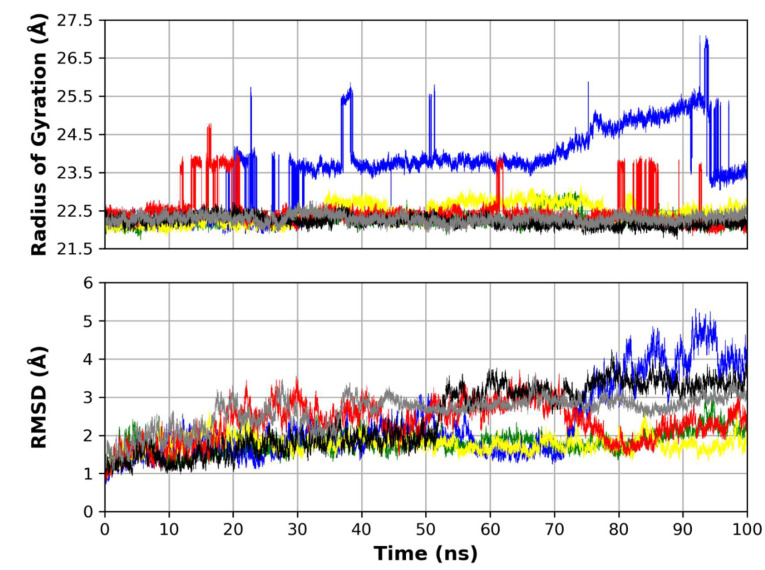
Top: R_g_ of the protein molecules in the six MD runs. Bottom: RMSD of the protein backbone atoms in the four MD runs. (Green: complex **1** (ferulic acid), blue: complex **2** (rutin), yellow: complex **3** (gallic acid), red: complex **4** (chlorogenic acid), black: complex **5** (co-crystallized N3), gray: complex **6** (docked N3)).

The radius of gyration (R_g_), a measure of protein compactness and equilibrium conformation, was measured for the four MD runs throughout the simulation time (Figure 2). The R_g_ of the Mpro complexed with both the co-crystallized N3 ligand and the docked N3 showed to be the least among all other simulations. This indicates the low conformational changes that took place in both runs and give the docked pose higher reliability due to its similar R_g_ to the co-crystallized one. The R_g_ of complexes **1** and **3** showed low fluctuation, while complexes **2** and **4** showed higher fluctuation with complex **2** having the highest R_g_ values throughout the simulation time. This can be attributed to a conformational change that took place in this complex. This can also be observed in the higher RMSD of protein atoms, and root mean square fluctuation (RMSF) of this complex protein residue, compared to other complexes.

The RMSD of the protein complex was measured in the six MD runs (Figure 2). The RMSD indicates the protein structure stability observed throughout the simulation time. The RMSD reached stability for the four MD runs with the highest RMSD for complex **2** followed by complex **4**. Both complexes **5** and **6** showed RMSD in relatively the same fashion as that of the other complexes, with a fluctuation in RMSD with an average of 4 Å, which is not considered a high fluctuation. This further indicates the stability of the other runs in comparison to the control simulation 5, and the validation of the docking pose 6 compared to the co-crystallized pose 5.

**Figure 3:**
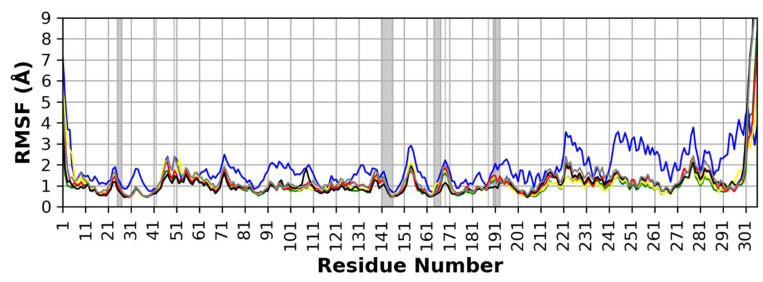
Per residue, RMSF for the protein residues in the six MD runs. (Green: complex **1** (ferulic acid), blue: complex **2** (rutin), yellow: complex **3** (gallic acid), red: complex **4** (chlorogenic acid), black: complex **5** (co-crystallized N3), gray: complex **6** (docked N3)). The binding site amino acids are highlighted with a grey background.

The per residue RMSF is a measure of protein residue conformational change and stability during the simulation. The RMSF values of the six MD runs are shown in (Figure 3). The binding site residues RMSF shows low RMSF during the simulation time for all complexes indicating their relatively strong binding to their respective bound ligands. Generally, complex **2** shows slightly higher RMSF than the other complexes, however, the RMSF of the key binding residues of the binding site show low RMSF corresponding to the strong binding to ligand **2** (rutin). The RMSF of complexes **5** and **6** show a similar pattern to the rest of the complexes which indicates a strong binding to the co-crystallized and docked N3 ligands, respectively.

Higher R_g_ and RMSF of complex **2** can suggest a conformational change rendered by the binding of compound **2** (rutin). This conformational change may be responsible for the inhibitory effect of rutin on the Mpro. However, this conformational change can only be determined by a longer simulation time. This can also encourage further study that correlates this major conformational change to its better activity.

**Figure 4:**
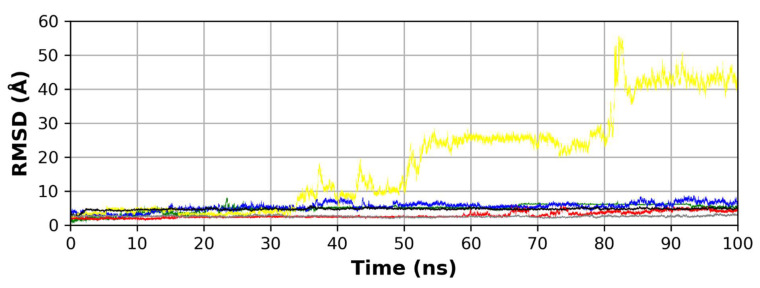
RMSD of each compound in the six MD runs. (Green: complex **1** (ferulic acid), blue: complex **2** (rutin), yellow: complex **3** (gallic acid), red: complex **4** (chlorogenic acid), black: complex **5** (co-crystallized N3), gray: complex **6** (docked N3)).

Ligand RMSD analysis during the simulation time shows that gallic acid **3** has the most deviation from its starting pose which indicates its weak binding to the protein and is confirmed by its whole abandonment off the binding site at the end of the simulation (Figure 4). Chlorogenic acid **4** shows the lowest RMSD during the simulation which reflects its good binding to the protein. Ferulic acid **1** and rutin **2** show relatively strong binding to the binding site which is reflected from their relatively low deviation during the simulation. Co-crystallized N3 **5** and docked N3 **6** show low deviation from their starting poses which indicates a good binding to the protein and the reliability of the docked pose as observed from its good agreement with the co-crystallized ligand indicated by the measures of Rg, protein RMSD and RMSF, ligand RMSD, and the number of hydrogen bonds.

**Figure 5:**
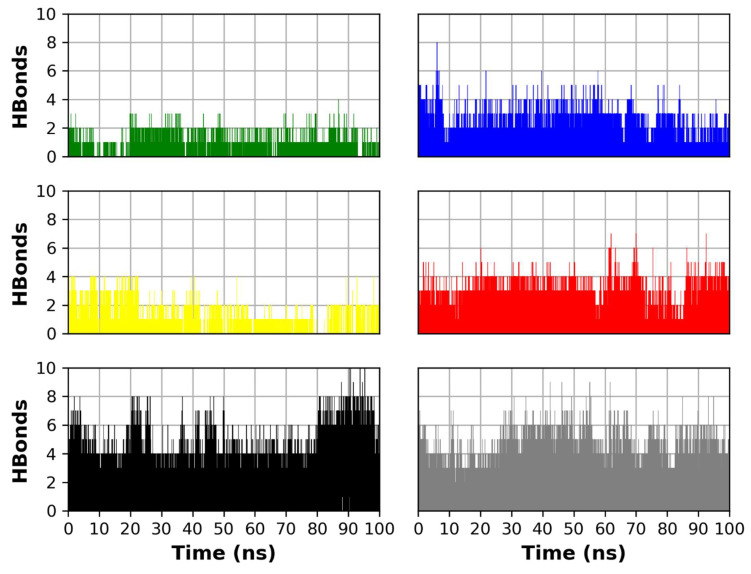
The number of H-bonds formed between each compound and the corresponding protein complex. (Green: complex **1** (ferulic acid), blue: complex **2** (rutin), yellow: complex **3** (gallic acid), red: complex **4** (chlorogenic acid), black: complex **5** (co-crystallized N3), gray: complex **6** (docked N3)).

Figure 5 describes the number of H-bonds formed between each compound and the protein during the simulation time. Compounds 5 and 6 show the highest number of H-bonds to the protein which is due to the higher number of functional groups present in them that possess anchorage sites for hydrogen bonding interactions. This also reflects the best docking score of the co-crystallized ligand (N3). On the other hand, ferulic acid **1** and gallic acid **3** show the least number of formed H-bonds with the protein indicating their weaker binding. Chlorogenic acid shows the highest number of formed hydrogen bonds 4, among the tested natural products, which can reflect its high activity in the biological evaluation and the docking experiment. Rutin 2 shows a relatively high number of H-bonds which also reflects its strong binding to the protein.

### 2.5. Assessment of In Vitro Cytotoxicity, Antiviral Activity, and Selectivity

The isolated compounds (ferulic acid **1**, rutin **2**, gallic acid **3**, and chlorogenic acid **4**) were tested for their cytotoxicity and virus-inhibitory effect by determining the half-maximal cytotoxic (CC_50_) and inhibitory (IC_50_) concentrations for each compound. The selectivity index for each of these compounds was determined as the ratio of the CC_50_ to the IC_50_.

**Figure 6:**
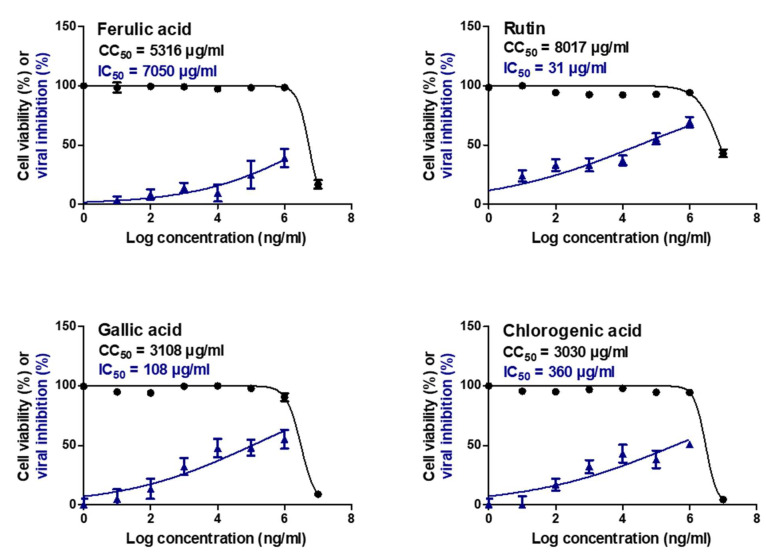
CC_50_ and IC_50_. The cytotoxicity values for studied compounds were assessed on Vero E6 cells while their antiviral activities were evaluated against SARS-CoV-2 (hCoV-19/Egypt/NRC-03/2020 (Accession Number on GSAID: EPI_ISL_430820).

Interestingly, rutin **2**, gallic acid **3**, and chlorogenic acid **4** showed promising antiviral activities against SARS-CoV-2 at IC_50_ values of 31 µg/mL, 108 μg/mL, and 360 µg/mL, respectively. Their selectivity indices were approximately 259, 29, and 8 (Figure 6 and Table 4), respectively.

With the lack of anti-SARS-CoV-2 activity of most FDA-approved protease inhibitors [36], lopinavir achieved moderate antiviral potential against SARS-CoV-2 at an IC_50_ value of 5.73 µM with a selectivity index of at least 8 [37]. Compared to lopinavir, we assume that rutin **2** and gallic acid **3** may be protease inhibitor candidates to combat SARS-CoV-2 replication.

Regarding the previously discussed findings concerning the activity of the tested compounds (ferulic acid **1**, rutin **2**, gallic acid **3**, and chlorogenic acid **4**) against SARS-CoV-2 between computational and in vitro insights showed that:

(a) The obtained docking results against SARS-CoV-2 Mpro as a proposed mechanism of their inhibitory action showed the following activity order (**2** > **4** > **1** > **3**). These results were further evaluated through molecular dynamics simulations which greatly supported the docking results.
(b) On the other hand, the in vitro anti-SARS-CoV-2 activities showed the following order (**2** > **3** > **4** > **1**) which may be attributed to the ability of gallic acid **3** to inhibit SARS-CoV-2 via more than one mechanism of action.
(c) Finally, these findings confirm the great impact of using computational tools to predict the biological activities of different compounds against various recommended targets.

### 2.6. Biochemical Studies and Interpretation

The results for six separate determinations were expressed as mean ± SD. With SPSS/18 Software, all the data were statistically evaluated. Statistical significance was considered for the *p* values of < 0.05.

Mercuric chloride (HgCl_2_) caused an apparent increase in the levels of TNF-α, IL-1β, and IL-2, indicating the production of inflammation. Previous studies have shown that heavy metals increase IL-1β, TNF-α, and IL-2 levels in lung tissue which may be similar to those produced in COVID-19 patients [38,39,40,41].

Table 5 revealed a significant elevation in plasma TNF-α, IL-1β, and IL-2 levels in HgCl_2_-treated rats compared to the normal control group (*p* < 0.05). The administration of *P. dioica* extract (50 mg/kg) and its isolated bioactive compounds [ferulic acid (50 mg/kg); rutin (75 mg/kg); gallic acid (50 mg/kg); chlorogenic acid (50 mg/kg.)] showed an apparent decrease in the levels of TNF-α, IL-1β, and IL-2 compared to HgCl_2_ treated group of rats after 15 days (*p* < 0.05). The effect of ferulic acid **1** and rutin **2** was pronounced more than the effect of total extract of *P. dioica*, gallic and chlorogenic acids.

In Table 6, an apparent increase serum Granulocyte-colony-stimulating factor (G-CSF) levels as well as an apparent decrease in the interleukin-10 (IL-10) levels were observed in the HgCl_2_-treated rats compared to the normal control group (*p* < 0.05), indicating acute lung inflammation. *P. dioica* extract (50 mg/kg b.w.) and its isolated bioactive compounds [ferulic acid (50 mg/kg b.w.); rutin (75 mg/kg b.w.); gallic acid (50 mg/kg b.w.); chlorogenic acid (50 mg/kg b.w.)] treatment significantly (*p* < 0.05) decreased serum G-CSF as well as significant increase in serum levels of IL-10 as compared to the HgCl_2_-treated group (*p* < 0.05).

**Figure 7:**
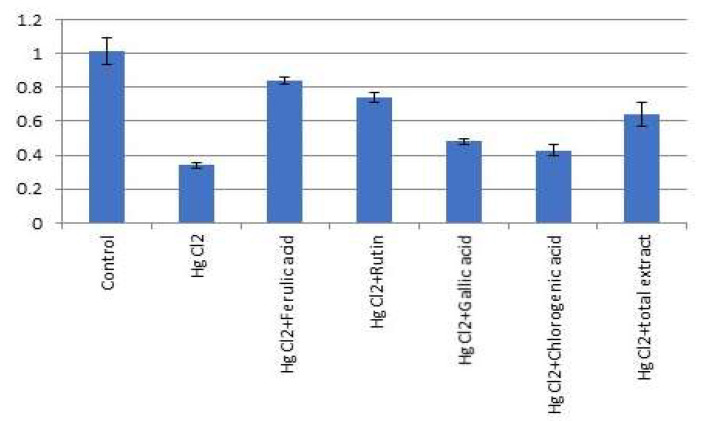
Effect of *P. dioica* aqueous extract and its isolated bioactive compounds on lung miRNA 21-3p expression in rats. Data (*n* = 10 per group) are presented as fold of control considering the normal control one.

**Figure 8:**
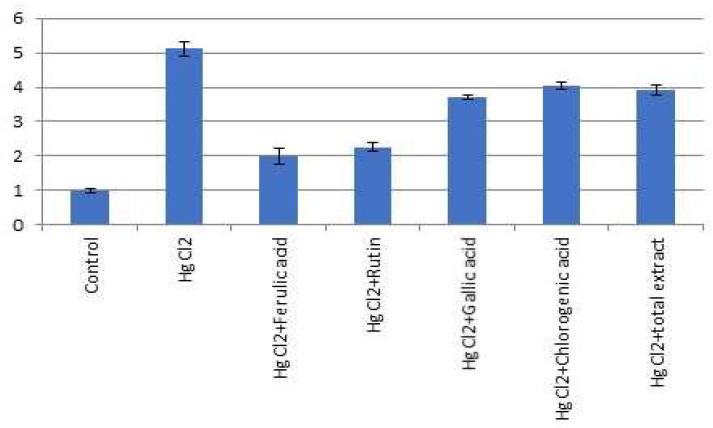
Effect of *P. dioica* aqueous extract and its isolated bioactive compounds on lung miRNA 155 expression in rats. Data (*n* = 10 per group) are presented as fold of control considering the normal control one.

Expression of miR-21-3p was significantly suppressed and miR-155 expression was significantly increased (*p* < 0.05) in HgCl_2_ (1.0 mg/kg) treated rats compared to the normal control group. Furthermore, *P. dioica* extract (50 mg/kg b.w.) and its isolated bioactive compounds [ferulic acid (50 mg/kg b.w.); rutin (75 mg/kg b.w.); gallic acid (50 mg/kg b.w.); chlorogenic acid (50 mg/kg b.w.)] treatment significantly increased (*p* < 0.05) the expression of miR-12-3p and suppressed miR-155 expression in the lungs of treated rats as compared to the HgCl_2_-treated group (Figure 7 and Figure 8).

The effect of ferulic acid **1** and rutin **2** was pronounced more than the effect of total extract of *P. dioica* as well as gallic and chlorogenic acids.

**Figure 9:**
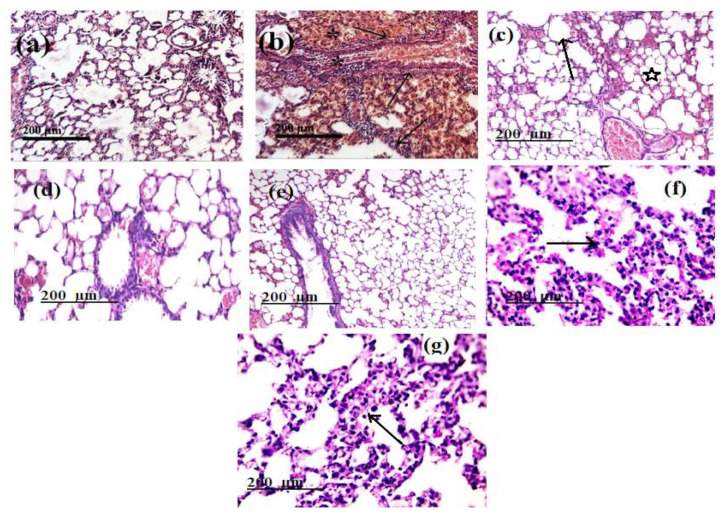
Sections stained with hematoxylin and eosin (H&E; 200 X) histological examination of rats’ lungs of different groups compared to control group (**a**) Negative control group (I); (**b**) Group II: positive control: (was received HgCl_2_ (1 mg/kg) for a 15-day period. (**c**) Group III: Was treated with HgCl_2_ (1 mg/kg.) + ferulic acid (50 mg/kg) for a 15-day period. (**d**) Group IV: Was treated with HgCl_2_ (1 mg/kg.) + rutin (75 mg/kg) for a 15-day period. (**e**) Group V: Was treated with HgCl_2_ (1 mg/kg.) + gallic acid (50 mg/kg) for a 15-day period; (**f**) Group VI: Was treated with HgCl_2_ (1 mg/kg.) + chlorogenic acid (50 mg/kg) for a 15-day period. (**g**): Group VII: Was treated with HgCl_2_ (1 mg/kg.) + *P. dioica* extract (50 mg/kg) for a 15-day period.

Histopathological examinations of normal Group (I) showing normal lung parenchyma (normal bronchi and pulmonary alveoli) (Figure 9a). Moreover, histological examination of the lung of the group of rats treated with HgCl_2_ group (II) showed significant divergent hemorrhagic pneumonia with blood extraversion, accompanied by severe thickening of the walls, peribronchiolar tissue with many inflammatory cells (arrows) (Figure 9b).

Histopathological examination also showed recovery of HgCl_2_-induced lung toxicity (III) by ferulic acid (50 mg/kg) compared to the HgCl_2_-treated group and produced nearly the same outputs as Groups I, (Figure 9c).

Group (IV and V) all samples of HgCl_2_ treated rats showed good recovery by treatment with rutin (75 mg/kg); gallic acid (50 mg/kg), respectively, and showing healthy lung parenchyma with the absence of collapse and normal bronchi and alveoli (Figure 9d,e). Group (VI and VII) all samples of HgCl_2_ treated rats showing improvement of the lung parenchyma; with the absence of collapse with moderate congestion in the peri-bronchial blood vessels by treatment with chlorogenic acid (50 mg/kg) and *P. dioica* extract (50 mg/kg), respectively (Figure 9f,g).

Herein, the administration of *P. dioica* extract and its isolated bioactive compounds ferulic acid **1**, rutin **2**, gallic acid **3**, and chlorogenic acid **4** showed a significant decrease in the levels of TNF-α, IL-1β, and IL-2 relative to HgCl_2_ treated group of rats.

A previous study using extracts of *P. dioca* found a reduction in the proinflammatory cytokines IL-6 and TNF-α [42,43]. *P. dioca*, has been demonstrated to regulate the inflammatory response when lung tissues were challenged with lipopolysaccharide (LPS) [44]. In the murine model, the inflammatory response was suppressed through the inhibition of TNF-α and IL-6. This was achieved via downregulation of the transcription factor nuclear factor-B. Furthermore, a previous study reported that the inhibitory effect of ferulic acid **1**, rutin **2**, gallic acid **3**, and chlorogenic acid **4** against inflammatory cytokines IL-6 and TNF-α [44,45,46]. It was stated previously that treatment with IL-2 can produce serious side effects including pulmonary edema [47].

In this study, increased serum G-CSF levels and IL-10 depletion could be attributed to increased production of reactive oxygen species, as evidenced by increased lipid peroxidation (LPO) levels following mercuric chloride treatment.

To assess *P. dioica* anti-inflammatory activity, we measured the anti-inflammatory cytokine G-CSF levels and IL-10. Interestingly, another study found that *P. dioica* reduced carrageenan-induced edema in a rat model, implying that it activates an anti-inflammatory pathway [48]. Other studies have shown that eucalyptol (one of the major compounds) in *P. dioica* reduced inflammation in carrageenan-induced paw edema in rats and mice [49,50]. IL-10 was demonstrated to inhibit pulmonary fibrosis development in TGF-B1 dependent manner. GCSF has been found to correlate with lung disease and induced airway inflammation and in response to cigarette smoke [51].

The fact that oral administration of ferulic acid **1**, rutin **2**, gallic acid **3**, and chlorogenic acid **4** significantly inhibited G-CSF and induced IL-10 formation during inflammation suggests that these compounds interfere with various aspects and mediators of inflammation. As a result, we propose that these isolates could act by inhibiting histamine release, prostaglandins produced by cyclooxygenase enzymes, lysosomal enzymes, and scavenging the free radicals from polymorphonuclear leucocytes, which would cause tissue damage at the inflammation site.

In this study, we found that mercuric chloride-inhibited lung miRNA21-3p and induced miRNA155 expression in rats.

MiR-155 is the most common amplifying of miRNAs during lung cancer and inflammation [52,53]. Moreover, several miRNAs for example miR-21-3p, miR-214, and miR-205 were down-regulated of non-small cell lung cancer (NSCLC) [54].

The present work suggested that *P. dioica* aqueous extract and its isolated bioactive compounds ferulic acid **1**, rutin **2**, gallic acid **3**, and chlorogenic acid **4** regulate mRNA 21-3P and 155 expressions via their anti-inflammatory activity against elevation of cytokines in mercuric chloride-treated rats.

In this study, HgCl_2_ administration is compatible with a previous study, with upregulated cytokine protein. The inductive inflammatory markers can attenuate the inflammatory response and oxygenation deficiency following the aspiration of HCl when blocked with phenolic compounds. Cytokines are expressed in lung and pulmonary endothelium and epithelium inflammatory cell residents and their induction causes an increased expression of prostanoids which play a key role in lung inflammatory modulation. Plant-derived polyphenols in this study significantly decreased cytokine protein confirming its anti-inflammatory activity that was previously reported [55]. Phenolic compounds were reported to decrease cytokine expression in different tissues and target miRNAs [56,57,58,59].

Serious lung histological changes in rats treated with HgCl_2_ were also observed. *P. dioica* extract and its isolated bioactive compounds treatment almost normalized these lung histoarchitecture effects. Also, the most important finding was that this model with *P. dioica* extract and its isolated bioactive compounds supplement reduced lung proliferation and oxidative stress, and inflammatory reactions with more pronounced effects in ferulic acid **1** and rutin **2**.

## 3. Materials and Methods

### 3.1. Plant

Leaves of *P. dioica* were collected in December 2019 from El Zohria Garden, Giza, Egypt. The used plant was authenticated by Dr. Ahmed Wahba, executive manager of the garden. The voucher sample (Pm.d-122019) was deposited in the herbarium of the Department of Pharmacognosy, Faculty of Pharmacy, October 6 University.

### 3.2. Animals

The Faculty of Veterinary Medicine, Benha University was the source of albino rats (males, weight = 195 ± 10 g). The animals were kept in plastic cages within a light-controlled room at 22 ± 1 °C and with a humidity of 55–60% for 1 week where they were supplied with a standard water and diet *ad libitum*. Before the experiment, the rats were categorized into 7 groups, each of 10 members. Group 1: received 5 mL distilled water. Group 2: obtained 1 mg HgCl_2_/kg [60]. Group 3: rats received 1mg HgCl_2_/kg + ferulic acid (50 mg/kg) [61]. Group 4: rats were provided with 1 mg HgCl_2_/kg + rutin (75 mg/kg) [62]. Group 5: rats received 1 mg HgCl_2_ mg/kg + gallic acid (50 mg/kg) [17]. Group 6: rats received 1 mg HgCl_2_/kg + chlorogenic acid (50 mg/kg) [63]. Group 7: rats received 1 mg HgCl_2_/kg + *P. dioica* aqueous extract (50 mg/kg). The treatment for all the groups was given daily for 15 days [64].

### 3.3. Virus, and Cells

Dulbecco’s modified Eagle’s medium containing 10% of fetal bovine serum, and 1% of Lonza (antimycotic antibiotic mixture) was used to grow Vero E6 cells. Vero E6 cells were kept at 37 °C and 5% CO_2_. An hCoV-19/Egypt/NRC-3/2020 SARS-CoV-2 virus (Accession Number on GSAID: EPI_ISL_430820) was cultivated in Vero E6 cells and harvested following the appearance of cytopathic effects (CPE).

### 3.4. Chemicals and Equipment

Standards for the RP-HPLC study were supplied from Central Lab in National Research Centre, silica gel 60 (Fluka), Sephadex LH20 was applied for column chromatography. The TLC was applied on silica gel pre-coated plates using the different solvents of highly commercial grades. Mercuric chloride was purchased from Sigma Chemical Co. (St.Louis, MO, USA).

NMR Instrument^: 1^H-NMR (400, MH_Z_) and ^13^C-NMR (100, MH_Z_) were measured on the Bruker High-Performance Digital FT-NMR spectrometer Avance III 400MH_Z_. The NMR spectra were measured in DMSO-*d6*. Mass spectrometer: EI/MS spectra were obtained on Thermo Scientific, ISO Single Quadrupole MS (USA).

## 4. Experimental Section

### 4.1. Extraction

The powder obtained from the air-dried leaves of *P. dioica* (1 kg) was exhaustively extracted with methanol (4 L). The combined MeOH extract was concentrated under reduced pressure to yield viscous residue (140 gm). The residue was then suspended in water (400 mL) and partitioned sequentially with dichloromethane (3 L) and ethyl acetate (3 L). The solvents were subjected to evaporation under reduced pressure to yield fractions of (8 g) and (17 g), respectively. The EtOAc fraction was subjected to the Si gel column and eluted with CH_2_Cl_2_-MeOH (100:0 and 20:80), giving three fractions (**1**–**3**) which were subjected to several runs using CH_2_Cl_2_-MeOH and n-hexane-ethyl acetate of different concentrations, similar fractions were pooled together using TLC. Sephadex LH-20 columns were applied for further isolation of four compounds; A (40 mg), B (60 mg), C (30 mg) and D (30 mg); respectively.

#### RP-HPLC Analysis

The aqueous leaves extract of *P. dioica* phenolic composition was investigated via RP-HPLC. The detailed methodology was inserted in the Supplementary Data (SI3).

### 4.2. Docking Studies

The MOE 2019.012 suite [65] was applied to carry out the docking studies for the isolated and identified four compounds (ferulic acid **1**, rutin **2**, gallic acid **3**, and chlorogenic acid **4**) of *P. dioica* (L.) Merr extract to propose their mechanism of action as SARS-CoV-2 Mpro inhibitors through evaluating their binding scores and modes compared to the co-crystallized inhibitor (N3) as a reference standard.

#### 4.2.1. The Isolated and Identified Four Compounds (**1**–**4**) Preparation

The 3D structures of the isolated identified four compounds (ferulic acid **1**, rutin **2**, gallic acid **3**, and chlorogenic acid **4**) were downloaded from the PubChem database and prepared for docking following the preparation steps described earlier [66,67,68]. Here, they were introduced into the MOE window, subjected to partial charges addition, and energy minimized [69,70]. Then, the prepared compounds were inserted into one database with the co-crystallized inhibitor (N3) and saved as an MDB file to be uploaded in the ligand icon during the docking step.

#### 4.2.2. The Target Mpro of SARS-CoV-2 Preparation

The X-ray of the target Mpro of SARS-CoV-2 was obtained from the Protein Data Bank (PDB code: 6LU7) [71]. Moreover, it was prepared for the docking process following the previously described steps in detail [72,73]. Notably, the downloaded protein was corrected for any errors, loaded with 3D hydrogens, and energy minimized as well [22,74].

#### 4.2.3. Docking of the Prepared Database to the Prepared Mpro of SARS-CoV-2

**Figure 10:**
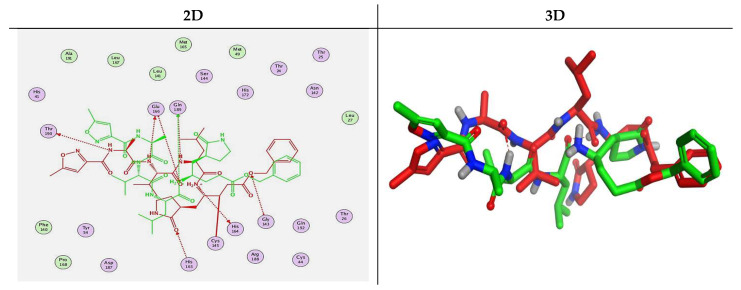
Superimposed poses of the docked N3 inhibitor (represented in green color) over the native co-crystallized one (represented in red color) produced from the redocking process inside the Mpro binding pocket. Left (**2D**) and right (**3D**) graphical representations.

The prepared database was inserted in a general docking process in place of the ligand site. The docking site was chosen to be the co-crystallized ligand site and the docking process was initiated after adjusting the default program specifications described before [75]. Briefly, the dummy atoms method was used to select the docking position. Triangle matcher and London dG were selected as the placement and the scoring methodologies, respectively. Both the refinement methodology and the scoring one were changed to the rigid receptor and GBVI/WSA dG, respectively, to extract the best 10 poses produced from 100 poses for each docked molecule [76,77]. The best pose for each ligand with the most acceptable score, binding mode, and RMSD value was selected for further studies. It is worth clarifying that a program validation step was performed first for the applied MOE program by redocking the co-crystallized native inhibitor (N3) at its binding pocket of the prepared main protease [78,79]. A valid performance was confirmed by obtaining a low RMSD value (1.43) between the native and redocked N3 ligands (Figure 10).

### 4.3. Molecular Dynamics Simulations

The previously used SARS-CoV-2 Mpro (PDB ID: 6LU7) was subjected to six runs of molecular dynamics simulations with the six ligands (ferulic acid **1**, rutin **2**, gallic acid **3**, chlorogenic acid **4**, co-crystallized N3 **5**, and docked N3 **6**). The simulations were performed at the NPT (constant number of molecules, pressure, and temperature). The detailed methodology for preparation was inserted in the Supplementary Data (SI4).

### 4.4. In Vitro Virological Studies

#### 4.4.1. In Vitro Cytotoxicity Assay

Ferulic acid **1**, rutin **2**, gallic acid **3**, and chlorogenic acid **4** were evaluated in vitro using the MTT method [80] with minor modification for their cytotoxicity in Vero E6 cells. Collectively, Vero E6 cells were grown in 96-well plates (cell density ≈ 1 × 10^4^/well) and kept in 5% CO_2_ for 24 h at 37 °C. On the second day, 1X PBS was used to wash the cells which were then subjected to different concentrations of ferulic acid **1**, rutin **2**, gallic acid **3**, and chlorogenic acid **4** (10 mg/mL to 1 ng/mL) in triplicates. Then, 72 h later, 20 µL of 5 mg/mL from the stock solution (MTT solution) was poured into each well and kept for 4 h at 37 °C followed by adding DMSO (200 µL) to dissolve the produced formazan crystals. Furthermore, the formazan solution absorbance was recorded with λ max 620 nm as a reference wavelength at 540 nm applying a reader of a multi-well plate. The untreated cells were used to calculate the cytotoxicity percentage. Graph Pad Prism 5 was used to plot the % cytotoxicity curve of each tested compound and CC_50_ (the half-maximal cytotoxic concentration) was extracted from the linear equation.

#### 4.4.2. Inhibitory Concentration 50 (IC_50_) Determination

To assess the anti-SARS-CoV-2 activity the IC_50_ values for the tested ingredients (**1**–**4**) were assayed as previously described [81]. Moreover, the exact methodology was described in the Supplementary Data (SI5).

### 4.5. Biochemical Assessments

Blood samples of every fasted rat have been obtained from the retro-orbital vein. Sodium fluoride as an anticoagulant was used, blood samples centrifuged, and plasma collected.

ELISA kits (Hengyuan Biotechnology Development Co., Ltd. Shanghai, China) and (Biolegend Systems, San Diego, CA, USA) were performed for plasma TNF-α, IL-1β, and IL-2, as well as for serum G-CSF and IL-10.

#### 4.5.1. RNA Extraction

The total RNA was isolated according to the manufacturer’s protocol (Ambion, Austin, TX, USA) from 200 μg tissue using the mirVana PARIS Kit. The exact methodology was described in the Supplementary Data (SI6).

#### 4.5.2. qRT-PCR of miRNAs 21-3p and 155

All samples have been executed three times. By melting curve analysis, the specificity of PCR products was measured. Expression levels were measured by the reverse transcript Kit (Takara Bio USA, Inc.), both miR-21-3p and miR-155, according to the manufacturer’s recommendations; quantitative PCRs (miRNA and mRNA) with green staining were detected. It was determined that relative expression [82], and GAPDH expression were used as internal controls. Also, the total methodology was described in the Supplementary Data (SI7).

Shanghai Sangon Biotech synthesized the primers for the detection of miR-21-3p and miR-155. The sequences subjected to PCR were: miRNA21-3p, forward 5′- CTCAACT-GGTGTCGTGGAGTCGGCAATTC-AGTTGAGGACAGCCC-3′, Reverse5′-ACACTCCAGC-TGGGCAACAGCAGTCGATGG-3′; miRNA-155, forward 5′- CGGCGGTTAATGCTAATTGTGAT-3′, reverse 5′-GTGCAGGGTCCGAGGT-3′; GAPDH forward, 5′-CATGAGAAGTAT- GACAACAGCCT-3′ and reverse, 5′-AGTCCTTCCACGATACCA AAGT-3′.

#### 4.5.3. Histological Assessment

The pieces produced from the lung cut were fixed for histological study by 10% buffered formaldehyde solution. For light microscopic analyses using the technique of Bancroft and Steven, sections with 5 mL thickness were prepared and then stained using hematoxylin and eosin [83].

## 5. Conclusions

Four isolates were extracted from the leaves of *P. dioica* (L.) Merr EtOAc extract (ferulic acid **1**, rutin **2**, gallic acid **3**, and chlorogenic acid **4**), and the results of RP-HPLC identified the major phenolics and flavonoids in the plant. The mechanism of action of the isolated compounds was proposed using both molecular docking and dynamics as SARS-CoV-2 Mpro inhibitors. In particular, rutin **2** achieved the best binding score (−9.19 Kcal/mol) compared to the docked co-crystallized inhibitor (−9.22 Kcal/mol) which was also confirmed by molecular dynamics simulations. Moreover, they were examined in vitro against SARS-CoV-2 using both cytotoxicity and inhibitory concentration 50 (IC_50_) determination studies. Interestingly, rutin **2**, gallic acid **3**, and chlorogenic acid **4** showed remarkable antiviral activities against SARS-CoV-2 at IC_50_ values of 31, 108, and 360 µg/mL, respectively. Additionally, the anti-inflammatory potentials of the *P. dioica* extract and its isolated bioactive compounds (**1**–**4**) were evaluated to examine their efficacy towards the cytokine storm. The results demonstrated their anti-inflammatory effects in HgCl_2_-treated rats through the reduction in the levels of TNF-α, IL-1β, IL-2, G-CSF, and mRNA155 gene expression. Furthermore, up-regulation of IL-10 levels, as well as mRNA21-3p gene expression, were observed in the lung-protective effects of *P. dioica* extract and its isolated bioactive compounds (**1**–**4**) against HgCl_2_ induced lung toxicity with better effects in ferulic acid **1** and rutin **2** treatments. The aforementioned results could be a good starting point for further optimization and for carrying out more advanced preclinical and clinical studies on the isolated compounds especially rutin **2** either alone or in combination with other isolates for COVID-19 control. Furthermore, these compounds as natural products may be examined alone or in combination with other natural or synthetic products as well.

## Figures and Tables

**Table 1:** RP-HPLC analysis of phenolics and flavonoids components of the aqueous leaves extract of *P. dioica*.

Identified Component	Area	Conc. (µg/g)
Gallic acid	3427.40	13,496.53
Chlorogenic acid	1095.38	3897.96
Catechin	621.52	3443.93
Methyl gallate	194.12	132.68
Caffeic acid	97.89	166.34
Syringic acid	80.70	180.96
Pyro catechol	109.82	343.14
Rutin	160.07	966.47
Ellagic acid	529.16	1797.51
Coumaric acid	641.32	557.62
Vanillin	ND	ND
Ferulic acid	322.33	520.92
Naringenin	2783.46	7514.91
Taxifolin	138.86	467.37
Cinnamic acid	ND	ND
Kaempferol	ND	ND

ND: not detected.

**Table 2:** The binding scores and interactions of the examined isolated four compounds from *P. dioica* (L.) Merr extract (**1**–**4**) and the docked N3 inhibitor (**5**) inside the binding pocket of SARS-CoV-2 Mpro.

No.	Isolated Compound	S ^a^	RMSD ^b^	Receptor Interactions	Distance (Å)
1	Ferulic acid	−5.35	1.81	Glu166/H-donor	2.98
2	Rutin	−9.19	1.81	His163/H-acceptor Glu166/H-donor Glu166/H-donor Phe140/H-donor Cys145/H-donor His41/H-pi	2.78 2.87 2.88 2.89 3.23 3.93
3	Gallic acid	−4.52	1.58	Leu141/H-donor Glu166/H-acceptor	2.82 3.03
4	Chlorogenic acid	−7.18	1.66	Asn142/H-donor His164/H-donor Arg188/H-acceptor Met165/H-donor	2.72 2.77 3.30 3.48
5	Docked N3	−9.22	1.84	Asn142/H-acceptor His164/H-donor Cys145/H-donor	3.08 3.28 3.37

**^a^ S**: the score of a ligand inside the binding pocket (Kcal/mol). **^b^ RMSD**: The Root Mean Squared Deviation between the predicted pose and the crystal structure.

**Table 3:** 3D pictures of the receptor interactions and positioning between the examined isolated four compounds from *P. dioica* (L.) Merr extract (**1**–**4**) and the docked N3 inhibitor (**5**) inside the binding site of SARS-CoV-2 Mpro. The red dash represents H-bonds and the black one represents H-pi interactions.

Isolated Comp.	3D Receptor Binding	3D Receptor Positioning
Ferulic acid (**1**)	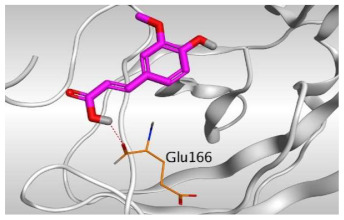	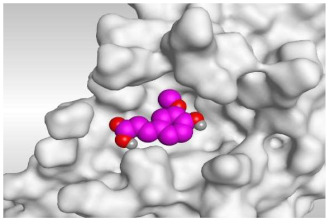
Rutin (**2**)	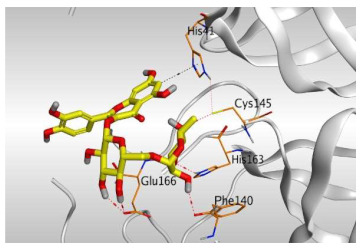	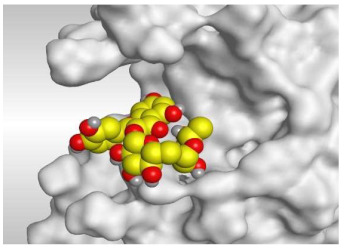
Gallic acid (**3**)	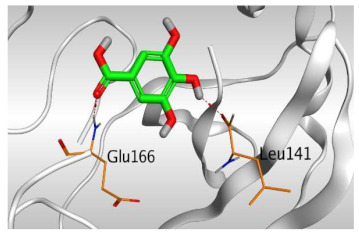	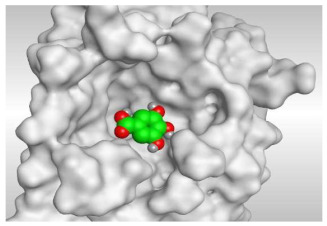
Chlorogenic acid (**4**)	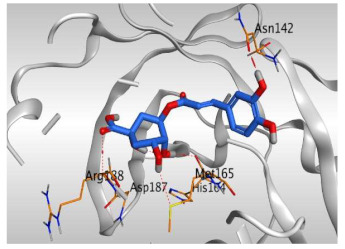	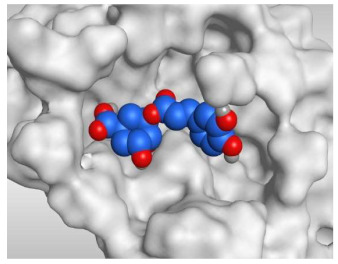
Docked N3 Inhibitor (**5**) *	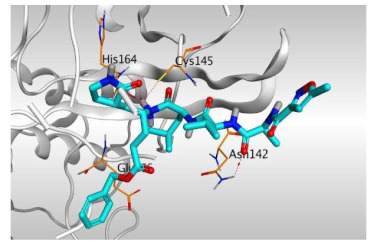	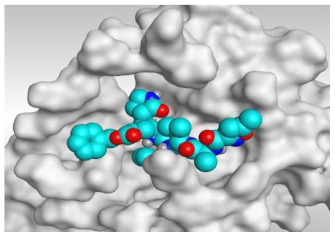

* The overlapping pose of the docked N3 inhibitor versus the co-crystallized one was presented in the Supplementary Data (SI2 and Figure S1).

**Table 4:** Cytotoxicity and virus-inhibitory effect of the isolated compounds (**1**–**4**) against SARS-CoV-2.

Compound	Name	CC_50_ (µg/mL)	IC_50_ (µg/mL)	Selectivity Index CC_50_/IC_50_
1	Ferulic acid	5316	7050	0.75
2	Rutin	8017	31	259
3	Gallic acid	3108	108	29
4	Chlorogenic acid	3030	360	8

**Table 5:** Effect of *P. dioica* aqueous extract and its isolated bioactive compounds on TNF-α, IL-1β, and IL-2 in rats.

No.	Groups	TNF-α (pg/mL)	(IL-1β) (pg/mL)	(IL-2) (pg/mL)
(I)	Normal (5 mL distilled water)	27.60 ± 4.76 ^a^	46.47 ± 6.49 ^a^	7.11 ±0.78 ^a^
(II)	Positive control HgCl_2_ (1 mg/kg b.w. per day in distilled water)	188.09 ±10.02 ^b^	175.26 ±7.98 ^b^	32.46 ±3.54 ^b^
(III)	HgCl_2_ (1 mg/kg) + Ferulic acid (50 mg/kg b.w.)	43.85 ± 6.29 ^c^	56.54 ± 4.85 ^c^	10.49 ± 2.11 ^c^
(IV)	HgCl_2_ (1 mg/kg) + Rutin (75 mg/kg b.w.)	53.54 ± 4.20 ^d^	67.43 ±6.01 ^d^	14.80 ±2.34 ^d^
(V)	HgCl_2_ (1 mg/kg) + Gallic acid (50 mg/kg b.w.)	70.77 ± 6.48 ^e^	89.50 ± 7.68 ^e^	19.04 ± 1.30 ^e^
(VI)	HgCl_2_ (1 mg/kg) + Chlorogenic acid (50 mg/kg)	92.70 ± 9.28 ^f^	132.17 ±9.85 ^f^	26.02 ± 4.31 ^f^
(VII)	HgCl_2_ (1 mg/kg) + *P. dioica* (50 mg/kg)	79.61 ± 6.97 ^g^	115.41 ± 7.88 ^g^	21.49 ±2.42 ^e^

Values represent the mean ± SE (*n* = 10). Data shown are mean ± standard deviation of the number of observations within each treatment. Data followed by the same letter (a, b, c, d, e, f, and g) within the same parameter are not significantly different at *p* ≤ 0.05.

**Table 6:** Effect of *P. dioica* aqueous extract and its isolated bioactive compounds on serum G-CSF and IL-10 in rats.

No.	Groups	G-CSF (pg/mL)	(IL-10) (pg/mL)
(I)	Normal (5 mL distilled water)	110.97 ± 11.31 ^a^	29.47 ±3.02 ^a^
(II)	Positive control HgCl_2_ (1 mg/kg b.w. per day in distilled water)	301.96 ± 17.07 ^b^	10.72 ± 1.81 ^b^
(III)	HgCl_2_ (1 mg/kg.) + Ferulic acid (50 mg/kg b.w.)	127.57 ± 9.05 ^c^	25.68 ± 3.08 ^c^
(IV)	HgCl_2_ (1 mg/kg.) + Rutin (75 mg/kg b.w.)	145.96 ± 8.64 ^d^	21.11 ± 1.45 ^d^
(V)	HgCl_2_ (1 mg/kg.) + Gallic acid (50 mg/kg b.w.)	176.23 ± 6.94 ^e^	17.67 ± 1.68 ^e^
(VI)	HgCl_2_ (1 mg/kg.) + Chlorogenic acid (50 mg/kg)	216.30 ± 13.81 ^f^	14.40 ± 1.24 ^f^
(VII)	HgCl_2_ (1 mg/kg.) + *P. dioica* (50 mg/kg)	201.33 ± 9.42 ^f^	15.72 ± 2.15 ^ef^

Values represent the mean ± SE (*n* = 10). Data shown are mean ± standard deviation of the number of observations within each treatment. Data followed by the same letter (a, b, c, d, e, and f) within the same parameter are not significantly different at *p* ≤ 0.05.

## Data Availability

Not applicable.

## Supplementary Materials

The following are available online, SI1: Chemistry of the isolated compounds, SI2: The overlapping poses of the co-crystallized versus the docked N3 inhibitor (Table 3), Figure S1: Superimposed poses of the docked N3 inhibitor (**5**) (represented in green color) over the native co-crystallized one (represented in red color) inside the Mpro binding pocket (from Table 3). Above (2D) and below (3D) graphical representations, SI3: Methodology of RP-HPLC analysis, SI4: Methodology of molecular dynamics simulations, SI5: Methodology of inhibitory concentration 50 (IC_50_) determination, SI6: Methodology of RNA extraction, and SI7: Methodology of qRT-PCR of miRNAs 21-3p and 155 are available online in the Supplementary Data file.

## Sample Availability

Samples of the compounds are available from the corresponding author.
