# Supplementary material for: Pimenta dioica (L.) Merr. Bioactive Constituents Exert Anti-SARS-CoV-2 and Anti-Inflammatory Activities: Molecular Docking and Dynamics, In Vitro, and In Vivo Studies
Source: Molecules. 2021 Sep 27;26(19):5844. doi: 10.3390/molecules26195844 (PMC8510437; doi:10.3390/molecules26195844)
Supplement: Supplementary file 1 [file molecules-26-05844-s001.zip › molecules-1356363-SI.pdf]

Pimenta dioica (L.) Merr. Bioactive Constituents Exert Anti-SARS-CoV-2 and Anti-Inflammatory Activities: Molecular Docking and Dynamics, *In Vitro*, and *In Vivo* Studies

Heba A. El Gizawy[a,ψ], Sylvia A. Boshra[b,ψ], Ahmed Mostafa[c], Sara H. Mahmoud[c], Muhammad I. Ismail[d], Aisha A. Alsfouk[e], Azza T. Taher[f,g,*], Ahmed A. Al-Karmalawy[h,*]

[a] Department of Pharmacognosy, Faculty of Pharmacy, October 6 University (O6U), October 6 city, Giza, Egypt. hebaelgizawy@o6u.com

[b] Department of Biochemistry, Faculty of Pharmacy, October 6 University (O6U), October 6 city, Giza, Egypt. Sylviaazmy@o6u.com

[c] Center of Scientific Excellence for Influenza Viruses, National Research Centre (NRC), Dokki, Giza 12622, Egypt. ahmed_elsayed@daad-alumni.de and Sarahussein9@yahoo.com

[d] Department of Pharmaceutical Chemistry, Faculty of Pharmacy, The British University in Egypt, Al-Sherouk City, Cairo-Suez Desert Road, 11837 Cairo, Egypt. m.ismail.800@gmail.com

[e] Department of Pharmaceutical Sciences, College of Pharmacy, Princess Nourah bint Abdulrahman University, Riyadh, Saudi Arabia. AAAlSfouk@pnu.edu.sa

[f] Department of Pharmaceutical Organic Chemistry, Faculty of Pharmacy, Cairo University, Cairo, Egypt.

[g] Department of Pharmaceutical Organic Chemistry, Faculty of Pharmacy, October 6 University (O6U), October 6 city, Giza, Egypt. azza.shalaby@pharma.cu.edu.eg

[h] Department of Pharmaceutical Medicinal Chemistry, Faculty of Pharmacy, Horus University-Egypt, New Damietta 34518, Egypt. akarmalawy@horus.edu.eg

Corresponding authors:

Ahmed A. Al-Karmalawy: akarmalawy@horus.edu.eg

Azza T. Taher: azza.shalaby@pharma.cu.edu.eg

ψ These authors equally contributed to this work.

Supplementary Information

SI1: Chemistry of the isolated compounds

Compound A was obtained as white powder, EI/MS showed molecular ion peak at m/z 194, ^{1}H-NMR (DMSO-d$_6$) δ ppm: 12.12 (1H, s, COOH), 9.55 (1H, s, OH), 7.09 (1H, dd, J = 8 and 1.3 Hz, H-6), 7.28 (1H, d, J = 1.3 Hz, H-2), 7.52 (1H,d, J = 15.8 Hz, H-1′), 6.39 (1H, d, J = 15.8 Hz, H-2′), 6.80 (1H,d, J = 8 Hz, H-5), 3.82 (3H, s, OCH3).^{13}C NMR (100 MHz, DMSO-d$_6$) δ 168.4 (C-3′), 149.5 (C-1), 148.3 (C-6), 144.9 (C-1′), 126 (C-4), 123.2 (C-3), 116 (C-2′), 115.9 (C-2), 111.6 (C-5), 56 (C-4′). Compound A was identified as ferulic acid[1].

Compound B was obtained as yellow powder, EI/MS showed molecular ion peak at m/z 610, ^{1}H-NMR (DMSO-d$_6$) δ ppm: 12.6 (s, OH), 7.55 assigned for H-2′/6′, 6.86 (1H, d, J= 8.4 Hz, H-2, H-5′), 6.39 (1H, d, J = 2 Hz, H-8), 6,20 (1H, d, J = 2 Hz, H-2, H-6), meta coupling between H6 and H8, signals of sugar protons appear at 5.36 (1H, d, J = 8 Hz, H-1′′), 4.35 (1H, d, J = 2Hz, H-1′′′), 3-3.7 (m, other sugar protons), 1 (3H, d, J = 6 Hz, methyl protons). Compound B was identified as rutin[2].

Compound C, white powder, ^{1}H-NMR (DMSO-d$_6$) δ ppm: 9.60 (1H, s, 3′OH), 9.2 (1H, s, 4′ OH), 7.4 (1H, d, J = 16 Hz, H-8′), 6.17 (1H, d, J = 16 Hz, H-7′), 7.04 (1H, d, J = 2Hz, H-2′), 6.99 (1H, dd, J = 8 and 2Hz, H-6′), 6.78 (1H, d, J = 8 Hz, H-5′), 5.08 (1H, q, H-5), 3.93 (1H, t, J = 3.3 Hz, H-4), 3.57 (1H, d, J = 4.5 Hz, H-3), 1.79–2.04 (4H, m, H-6, H-2), 1.81 (1H, m, H-2ax), 1.76 (1H, m, H-2eq). ^{13}C NMR (100 MHz, DMSO-d$_6$) δ 175.4 (C-7), 166.2 (C-9′), 148.8 (C-4′), 146 (C-7′), 145.4 (C-3′), 126 (C-1′), 121.8 (C-6′), 116.2 (C-5′), 115.2 (C-2′), 114.7 (C-8′), 73.9 (C-1), 70.8 (C-4), 71.3 (C-5), 68.5 (C-3), 37.6 (C-6), 36.7 (C-2). Compound C was identified as chlorogenic acid[3].

Compound D, buff powder, ^{1}H-NMR (DMSO-d$_6$) δ ppm: 6.92 (H-1), 9.19 (H-2), 8.83 (H-3), 12.23 (H-4), ^{13}CNMR (100 MHz, DMSO-d$_6$) δ 120.4 (C-1), 108 (C-2, C-6), 146 (C-3, C-5), 139 (C-4), 170 (C-7). Compound D was identified as gallic acid[4].

SI2: The overlapping poses of the co-crystallized versus the docked N3 inhibitor (Table 3).

2D
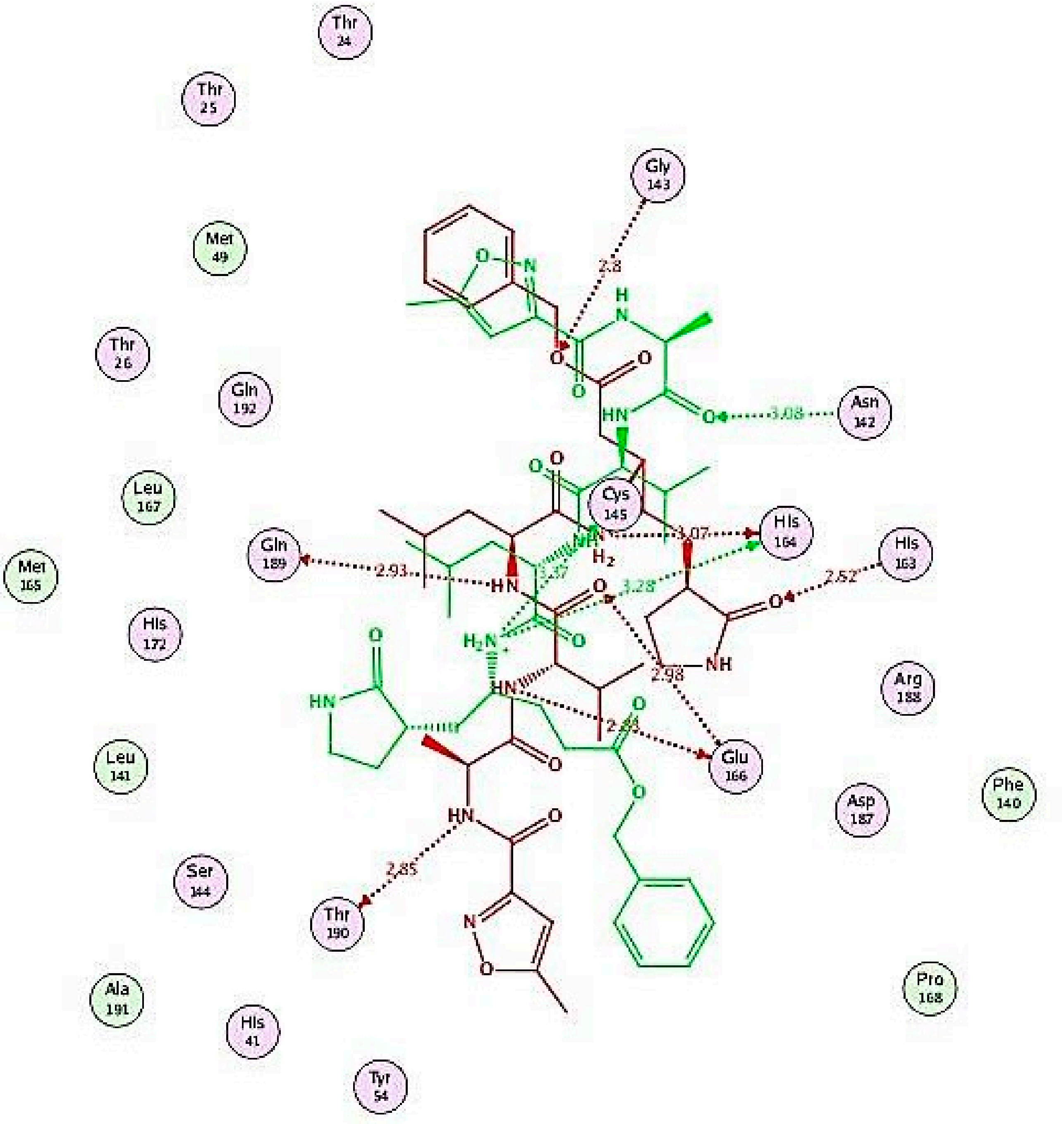

3D

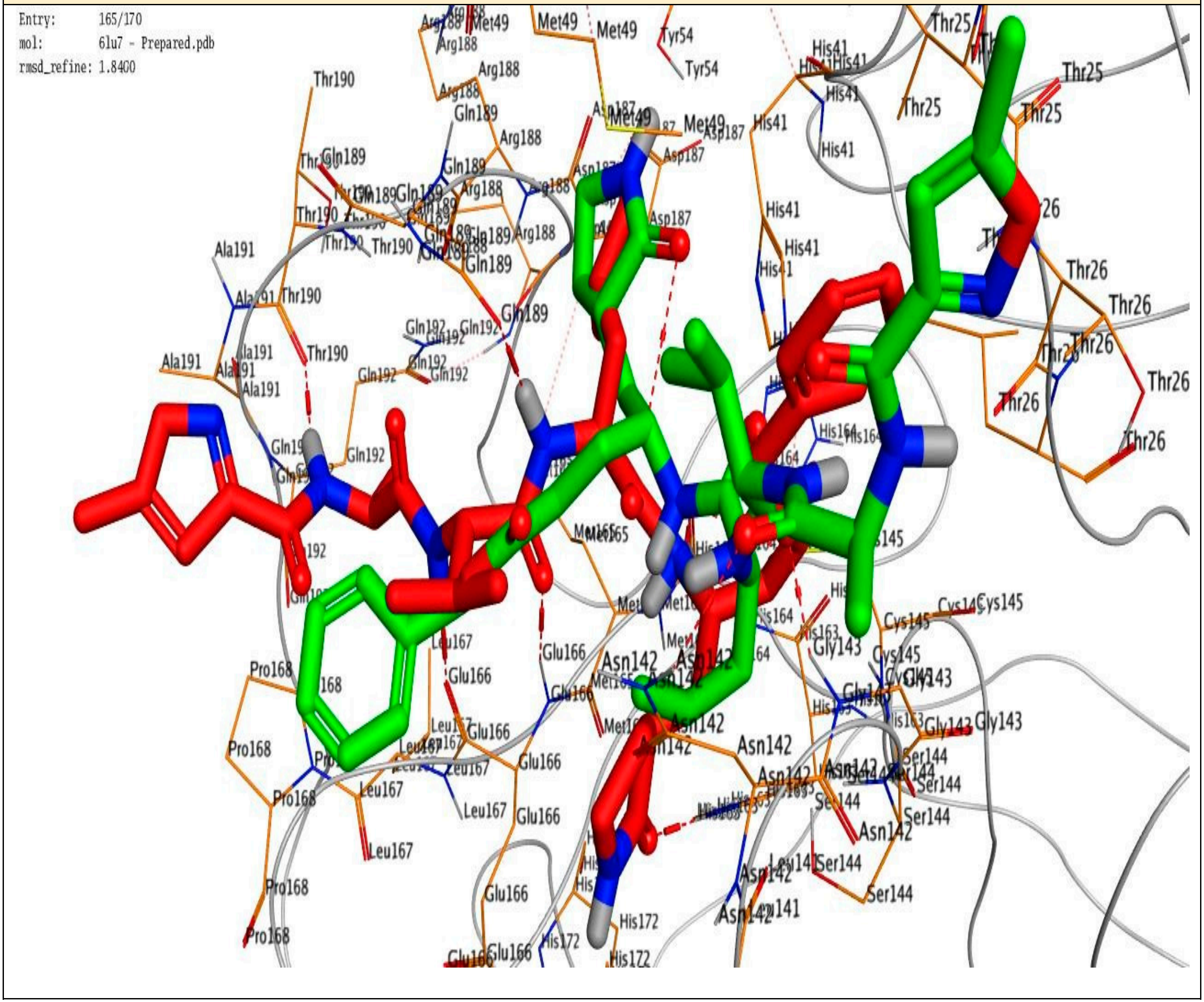

Figure S1. Superimposed poses of the docked N3 inhibitor (5) (represented in green color) over the native co-crystallized one (represented in red color) inside the Mpro binding pocket (from **Table 3**). Above (2D) and below (3D) graphical representations.

SI3: Methodology of RP-HPLC analysis

Agilent 1260 series was used to carry out the analysis. The Eclipse C18 column (4.6 mm x 250 mm i.d., 5 μm) was applied to perform the separation. The mobile phase with a 1 ml/min flow rate and composed of water (A) and 0.05% trifluoroacetic acid in acetonitrile (B). It was programmed consecutively in a linear gradient as follows: 0 min (82% A); 0–5 min (80% A); 5-8 min (60% A); 8-12 min (60% A); 12-15 min (85% A) and 15-16 min (82% A). The multi-wavelength detector was monitored at 280 nm. The injection volume (10 μl) was applied for each of the sample solutions and the temperature of the column was maintained at 35 °C.

SI4: Methodology of molecular dynamics simulations

The simulation system was set up using the OpenMM setup utility[5] by cleaning up the protein-ligand complex PDB file and adding missing atoms. Missing hydrogen atoms were added at PH 7.0, then the complex was immersed in a cubic water box with a padding distance of 1 nm along each axis of the complex molecule. NaCl molecules were added at a concentration of 0.15 Molar to neutralize the complex charges, with the system of the complex ending up with around 63,000 atoms. Protein molecule was described using AMBER 14 force field[6], and TIP3P-FB model[7] was used for describing water molecules. Generalized Amber Force Field (GAFF)[8] was used for small molecule parameters. GAFF parameters for small molecules were generated by on-the-fly template generation utilizing OpenEye toolkits 2019.4.1. ELF10 charges were assigned to small molecules using the canonical AM1-BCC charging method of OpenEye toolkits.[9] Long-range electrostatics were calculated using the Particle Mesh Ewald (PME) method[10] assigning the error tolerance for truncating the Ewald summation to 0.0005. The cut off for both PME direct space interactions and Lennard-Jones interactions was set to 1.2 nm. The length of all bonds that involve a hydrogen atom, and the water molecules, involving their bond length and angles, were constrained. Force equations were integrated using a Langevin integrator with 2 fs step size, 310 K temperature, and 1 ps^{-1} friction coefficient. Pressure coupling was maintained using a Monte Carlo barostat[11] assigning a constant temperature of 310 K, a

constant pressure of 1 atm, and a pressure update every 25 steps (50 fs). OpenMM 7.4.2 application layer was used for carrying out the dynamic simulations. The simulation started with energy minimization with 10 kJ/mole as energy convergence criteria, then equilibration for 500,000 steps (1 ns), and finally production run for 50,000,000 steps (100 ns). Trajectory reporting was set to one snapshot per 1000 steps resulting in 50,000 snapshots in the final trajectory file. RMSD, RMSF, and radius of gyration were calculated using ProDy python library[12, 13]. Ligand RMSD and number of hydrogen bonds to the protein were calculated using VMD RMSD trajectory analysis and hydrogen bonds utilities, respectively.[14] Figures were generated using matplotlib python library[15].

SI5: Methodology of inhibitory concentration 50 (IC_{50}) determination

In 96-well tissue culture plates, 2.4×10^4 Vero E6 cells were distributed in each well and incubated at 37 °C and 5% CO_2 for 24 h. The cell monolayers were then washed once with 1x PBS and subjected to hCoV-19/Egypt/NRC-03/2020 SARS-CoV-2 virus adsorption (100 TCID50) for 1 h at room temperature (RT). The cell monolayers were further overlaid with 50 μL of DMEM containing varying safe concentrations ($<CC_{50}$) of each tested compound (1-4) in triplicate. Following incubation at 37 °C in a 5% CO_2 incubator for 72 h, the cells were fixed with 100 μL of 4% paraformaldehyde for 20 min and stained with 0.1% crystal violet in distilled water for 15 min at RT. The crystal violet dye was then dissolved using 100 μL absolute methanol per well and the optical density of the color was measured at 570 nm using Anthos Zenyth 200rt plate reader (Anthos Labtec Instruments, Heerhugowaard, Netherlands). A control was included as an untreated virus in each plate (triplicate). The viral inhibition percentage curve of each tested compound (1-4) against SARS-CoV-2 was plotted using Graph Pad Prism 5 and the IC_{50} (50% inhibitory concentration) was calculated from the non-linear regression curve-fit analysis, relative to the virus and cell controls.

SI6: Methodology of RNA extraction

Each sample was mixed to denaturation solution and then spiked with 5 µl synthetic *C. elegans* miRNAs 21-3p and 155 (5 nM/L, Ribobio, Guangzhou, China) to normalize sample-to-sample variation. Following the instructions of the producer, total RNA was isolated by TRIzol (Invitrogen, Carlsbad, CA, USA). The RNA total was eluted with RNase-free water for 100 µl and stored for further analysis at −80°C. The Nanodrop 2000 spectrophotometer was used to determine RNA concentration and purity (NanoDrop Technologies, Wilmington, DE, USA).

SI7: Methodology of qRT-PCR of miRNAs 21-3p and 155

SYBR Green (SYBR® Premix Ex TaqTM II, TaKaRa, Dalian, China) has been used for the determination of miRNAs expression levels 21-3p and 155. The amplification of a single miRNA was performed with a Bulge-LoopTM miRNA qRT-PCR Primer Set (RiboBio, Guangzhou, China) which contained reverse transcription (RT) and PCR[16, 17] specific prime modules.

As described above, the RT reactions were carried on at 42 °C for 60 min, followed by 70 °C for 10 min, and the qRT-PCR reactions were carried on at 95 °C for 20 s, followed by 40 cycles of 95 °C for 10 s, 60 °C for 20 s, and then 70 °C for 10 s on a Light Cycler® 480 Real-Time PCR System (Roche Diagnostics, Mannheim, Germany) in 384-well plates[18].

References

1. Sajjadi, S. E.; Shokoohinia, Y.; Moayedi, N.-S., Isolation and Identification of Ferulic Acid From Aerial Parts of Kelussia odoratissima Mozaff. *Jundishapur journal of natural pharmaceutical products* **2012,** *7* (4), 159.
2. Fathiazad, F.; Delazar, A.; Amiri, R.; Sarker, S. D., Extraction of flavonoids and quantification of rutin from waste tobacco leaves. *Iranian Journal of Pharmaceutical Research* **2010,** (3), 222-227.
3. Kaiser, N.; Birkholz, D.; Colomban, S.; Navarini, L.; Engelhardt, U. H., A new method for the preparative isolation of chlorogenic acid lactones from coffee and model roasts of 5-caffeoylquinic acid. *Journal of agricultural and food chemistry* **2013,** *61* (28), 6937-6941.
4. Tukiran, T.; Mahmudah, F.; Hidayati, N.; Shimizu, K., GALLIC ACID: A PHENOLIC ACID AND ITS ANTIOXIDANT ACTIVITY FROM STEM BARK OF CHLOROFORM EXTRACTS OF SYZYGIUM LITORALE (BLUME) AMSHOFF (MYRTACEAE). *Molekul* **2016,** *11* (2), 180-189.
5. Eastman, P.; Swails, J.; Chodera, J. D.; McGibbon, R. T.; Zhao, Y.; Beauchamp, K. A.; Wang, L.-P.; Simmonett, A. C.; Harrigan, M. P.; Stern, C. D., OpenMM 7: Rapid development of high performance algorithms for molecular dynamics. *PLoS computational biology* **2017,** *13* (7), e1005659.
6. Maier, J. A.; Martinez, C.; Kasavajhala, K.; Wickstrom, L.; Hauser, K. E.; Simmerling, C., ff14SB: improving the accuracy of protein side chain and backbone parameters from ff99SB. *Journal of chemical theory and computation* **2015,** *11* (8), 3696-3713.
7. Wang, L.-P.; Martinez, T. J.; Pande, V. S., Building force fields: An automatic, systematic, and reproducible approach. *The journal of physical chemistry letters* **2014,** *5* (11), 1885-1891.
8. Wang, J.; Wolf, R. M.; Caldwell, J. W.; Kollman, P. A.; Case, D. A., Development and testing of a general amber force field. *Journal of computational chemistry* **2004,** *25* (9), 1157-1174.
9. Ismail, M. I.; Ragab, H. M.; Bekhit, A. A.; Ibrahim, T. M., Targeting multiple conformations of SARS-CoV2 Papain-Like Protease for drug repositioning: An in-silico study. *Computers in biology and medicine* **2021,** *131*, 104295.
10. Essman, U.; Perera, L.; Berkowitz, M.; Darden, T.; Lee, H.; Pedersen, L., A smooth particle mesh ewald potential. *J. Chem. Phys* **1995,** *103*, 8577-8592.
11. Chow, K.-H.; Ferguson, D. M., Isothermal-isobaric molecular dynamics simulations with Monte Carlo volume sampling. *Computer physics communications* **1995,** *91* (1-3), 283-289.
12. Bakan, A.; Meireles, L. M.; Bahar, I., ProDy: protein dynamics inferred from theory and experiments. *Bioinformatics* **2011,** *27* (11), 1575-1577.
13. Bakan, A.; Dutta, A.; Mao, W.; Liu, Y.; Chennubhotla, C.; Lezon, T. R.; Bahar, I., Evol and ProDy for bridging protein sequence evolution and structural dynamics. *Bioinformatics* **2014,** *30* (18), 2681-2683.
14. Humphrey, W.; Dalke, A.; Schulten, K., VMD: visual molecular dynamics. *Journal of molecular graphics* **1996,** *14* (1), 33-38.
15. Hunter, J. D., Matplotlib: A 2D graphics environment. *IEEE Annals of the History of Computing* **2007,** *9* (03), 90-95.
16. Zhou, X.; Zhu, W.; Li, H.; Wen, W.; Cheng, W.; Wang, F.; Wu, Y.; Qi, L.; Fan, Y.; Chen, Y., Diagnostic value of a plasma microRNA signature in gastric cancer: a microRNA expression analysis. *Scientific reports* **2015,** *5* (1), 1-13.
17. Zhu, M.; Huang, Z.; Zhu, D.; Zhou, X.; Shan, X.; Qi, L.-W.; Wu, L.; Cheng, W.; Zhu, J.; Zhang, L., A panel of microRNA signature in serum for colorectal cancer diagnosis. *Oncotarget* **2017,** *8* (10), 17081.
18. Livak, K. J.; Schmittgen, T. D., Analysis of relative gene expression data using real-time quantitative PCR and the 2− ΔΔCT method. *methods* **2001,** *25* (4), 402-408.
